# Myeloid Clusters Are Associated with a Pro-Metastatic Environment and Poor Prognosis in Smoking-Related Early Stage Non-Small Cell Lung Cancer

**DOI:** 10.1371/journal.pone.0065121

**Published:** 2013-05-24

**Authors:** Wang Zhang, Sumanta K. Pal, Xueli Liu, Chunmei Yang, Sachin Allahabadi, Shaira Bhanji, Robert A. Figlin, Hua Yu, Karen L. Reckamp

**Affiliations:** 1 Department of Cancer Immunotherapy and Immunology, City of Hope Comprehensive Cancer Center, Duarte, California, United States of America; 2 Department of Medical Oncology and Therapeutics Research, City of Hope Comprehensive Cancer Center, Duarte, California, United States of America; 3 Department of Information Sciences, City of Hope Comprehensive Cancer Center, Duarte, California, United States of America; 4 Department of Bioengineering, Rice University, Houston, Texas, United States of America; 5 Department of Economics, Harvard University, Cambridge, Massachusetts, United States of America; 6 Department of Medicine, Cedars Sinai Medical Center, Los Angeles, California, United States of America; Istituto Superiore di Sanità, Italy

## Abstract

**Background:**

This study aimed to understand the role of myeloid cell clusters in uninvolved regional lymph nodes from early stage non-small cell lung cancer patients.

**Methods:**

Uninvolved regional lymph node sections from 67 patients with stage I–III resected non-small cell lung cancer were immunostained to detect myeloid clusters, STAT3 activity and occult metastasis. Anthracosis intensity, myeloid cluster infiltration associated with anthracosis and pSTAT3 level were scored and correlated with patient survival. Multivariate Cox regression analysis was performed with prognostic variables. Human macrophages were used for *in vitro* nicotine treatment.

**Results:**

CD68^+^ myeloid clusters associated with anthracosis and with an immunosuppressive and metastasis-promoting phenotype and elevated overall STAT3 activity were observed in uninvolved lymph nodes. In patients with a smoking history, myeloid cluster score significantly correlated with anthracosis intensity and pSTAT3 level (P<0.01). Nicotine activated STAT3 in macrophages in long-term culture. CD68^+^ myeloid clusters correlated and colocalized with occult metastasis. Myeloid cluster score was an independent prognostic factor (P = 0.049) and was associated with survival by Kaplan-Maier estimate in patients with a history of smoking (P = 0.055). The combination of myeloid cluster score with either lymph node stage or pSTAT3 level defined two populations with a significant difference in survival (P = 0.024 and P = 0.004, respectively).

**Conclusions:**

Myeloid clusters facilitate a pro-metastatic microenvironment in uninvolved regional lymph nodes and associate with occult metastasis in early stage non-small cell lung cancer. Myeloid cluster score is an independent prognostic factor for survival in patients with a history of smoking, and may present a novel method to inform therapy choices in the adjuvant setting. Further validation studies are warranted.

## Introduction

Lung cancer is the most frequent cause of cancer death in the United States.[1] Despite reduced smoking rates, cigarette smoke is still the main risk factor associated with lung cancer.[2] Tobacco smoke contains thousands of chemicals, about 70 of which are known carcinogens.[3] Anthracosis, which is the deposition of black dust matter, has been found in the lungs and lymph nodes (LNs) of those with a history of smoking.[4] Exposure to tobacco smoke induces mutagenesis leading to the development of lung cancer, and continued smoking causes increased mortality and recurrence in early stage disease.[5] Although therapies are available for the treatment of non-small cell lung cancer (NSCLC), most patients develop recurrence due to its highly invasive and metastatic capacity. New strategies are needed for the early prediction of micrometastatic disease and invasive capacity of NSCLC.

Within the tumor microenvironment, immune cells produce immunosuppressive factors, such as interleukin (IL)-6, IL-10, transforming growth factor-β and vascular endothelial growth factor (VEGF), resulting in ineffective anti-tumor immune responses and promotion of tumor growth, angiogenesis and invasion.[6], [7], [8], [9], [10] The microenvironment of malignancies including NSCLC converts myeloid cells, which are essential for both innate and adaptive immunity, into immunosuppressive cells that facilitate immune evasion.[6], [11], [12], [13] Constitutive activation of signal transducer and activator of transcription 3 (STAT3) in myeloid cells is crucial for the development of immunosuppression in primary tumors and their microenvironment.[7], [9], [14]

The importance of myeloid cells has been described in conditioning pre-metastatic tissue for the future seeding of metastatic tumor cells.[15], [16], [17], [18], [19], [20], [21], [22], [23] In some tumor models, infiltrating myeloid cells in pre-metastatic tissue form clusters, which are so-called “pre-metastatic niches.”[16], [19], [20] In a recent report, the sphingosine-1-phosphate receptor 1 (S1PR1)-STAT3 signaling axis in myeloid cells was shown to be critical for myeloid cell colonization in pre-metastatic sites, promoting metastasis.[24] The correlation between infiltration of myeloid cell clusters at pre-metastatic sites and patient prognosis has not been previously described. We hypothesized that infiltration of myeloid cells and elevated STAT3 activity within pre-metastatic tissue from NSCLC patients could predict patient outcomes following surgical resection. Regional LNs are commonly invaded by NSCLC cells in the process of metastasis. We studied uninvolved regional LNs from patients with resectable NSCLC to determine the association between myeloid cell clusters, the activation of STAT3 and patient prognosis.

## Materials and Methods

### Clinical samples

Patient specimens were collected in accordance with the City of Hope institutional review board (IRB#10062) and patient written informed consent was obtained. Uninvolved lymph node tissue was collected from 67 patients with a pathologically verified diagnosis of NSCLC who underwent either mediastinal lymph node sampling or complete lymphadenectomy and were found to have pathologically determined N0 disease, or N1–N3 disease. The patients had at least 1 lymph node characterized as uninvolved with tumor, with available paraffin-embedded tissue. One uninvolved lymph node tissue block from each qualified patient was obtained and four-μm sections were prepared for subsequent analyses. Staging was determined by the American Joint Committee on Cancer Guidelines, 7^th^ edition.[25] Patient demographics and clinical characteristics are presented in Table 1. Normal LN sections from 4 individuals without cancer were purchased from Abcam, Imgenex and GeneTex, and all were ethically obtained and examined and diagnosed by licensed pathologists.

**Table 1:** Patient demographics and tumor characteristics.

Demographic or characteristic	Smoking group (n = 49)		Non-smoking group (n = 18)		P
	No. of patients	%	No. of patients	%	
Age at diagnosis, years					0.69
Median	71		65.5		
Range	45–85		49–86		
Gender					0.02
Male	22	44.9	2	11.1	
Female	27	55.1	16	88.9	
Ethnic origin					0.0006
White	46	93.9	10	55.6	
Asian	2	4.1	7	38.9	
Black	1	2.0	1	5.6	
Time of follow-up, months					0.056
Median	53.3		34.3		
Range	2.1–201.7		1.9–108.2		
ECOG					0.19
0	22	44.9	12	66.7	
1	27	55.1	6	33.3	
Smoking history			NA		NA
Current	20	40.8			
Former	29	59.2			
Tumor histology					0.18
Squamous cell carcinoma	15	30.6	2	11.1	
Adenocarcinoma	30	61.2	16	88.9	
Large cell carcinoma	2	4.1	0	0.0	
Mixed carcinoma	2	4.1	0	0.0	
Pathologic stage					
Overall stage					0.49
I	23	46.9	10	55.6	
II	12	24.5	2	11.1	
III	14	28.6	6	33.3	
T stage					0.75
T1	18	36.7	9	50.0	
T2	23	46.9	6	33.3	
T3	6	12.2	2	11.1	
T4	2	4.1	1	5.6	
N stage					0.78
N0	28	57.1	12	66.7	
N1	11	22.5	3	16.6	
N2	10	20.4	3	16.6	
Average No. of mediastinal LNs sampled	5.1		4.5		0.24
Pre-surgical therapy received					0.96
None	44	89.8	16	88.9	
Chemotherapy	2	4.1	1	5.6	
Radiotherapy	0	0.0	0	0.0	
Chemoradiotherapy	3	6.1	1	5.6	
Post-surgical therapy received					0.41
None	31	63.3	14	77.7	
Chemotherapy	3	6.1	2	11.1	
Radiotherapy	9	12.2	1	5.6	
Chemoradiotherapy	6	18.4	1	5.6	

NA, not applicable.

### Immunohistochemistry (IHC)

Sections were incubated with antibodies against CD68 (AbD Serotec, clone 514H12, 1∶50), pY705-STAT3 (Cell Signaling, clone D3A7, 1∶50), CD33 (Leica, clone PWS44, 1∶200), CD163 (AbD Serotec, clone EDHu-1, 1∶200), IL-6 (Abcam, 1∶400), IL-10 (AbD Serotec, clone JES3-12G8, 1∶100), VEGF-A (Abcam, clone VG-1, 1∶200), matrix metalloproteinase 9 (MMP-9, Cell Signaling, 1∶100), Stromal cell-derived factor 1/Chemokine (C-X-C motif) ligand 12 (SDF-1/CXCL12, R&D, clone 79018, 1∶100), B-cell lymphoma-extra large (Bcl-xL, Cell Signaling, clone 54H6, 1∶200) or pan-cytokeratin (pan-CK, eBioscience, clone AE1/AE3 [26], [27], 1∶1000) overnight at 4°C. ABC elite kit and 3,3′-Diaminobenzidine (Vector Labs) were used according to specifications. Images were captured with Nikon Eclipse TE2000-U microscope. Quantification was performed with Image Pro Plus.

### Immunofluorescence and confocal microscopy

After antigen retrieval, sections were blocked with Image-iT FX Signal Enhancer (Invitrogen) followed by 10% goat serum, and incubated with primary antibodies overnight at 4°C. Cellular fibronectin (cFn) antibody was from Abcam. Slides were then probed with either fluorophore-labeled secondary antibody or biotinylated secondary antibody followed by fluorophore-labeled streptavidin. Images were captured with Zeiss LSM510 upright confocal microscope.

### Scoring method and the detection of occult metastasis

**Figure 1:**
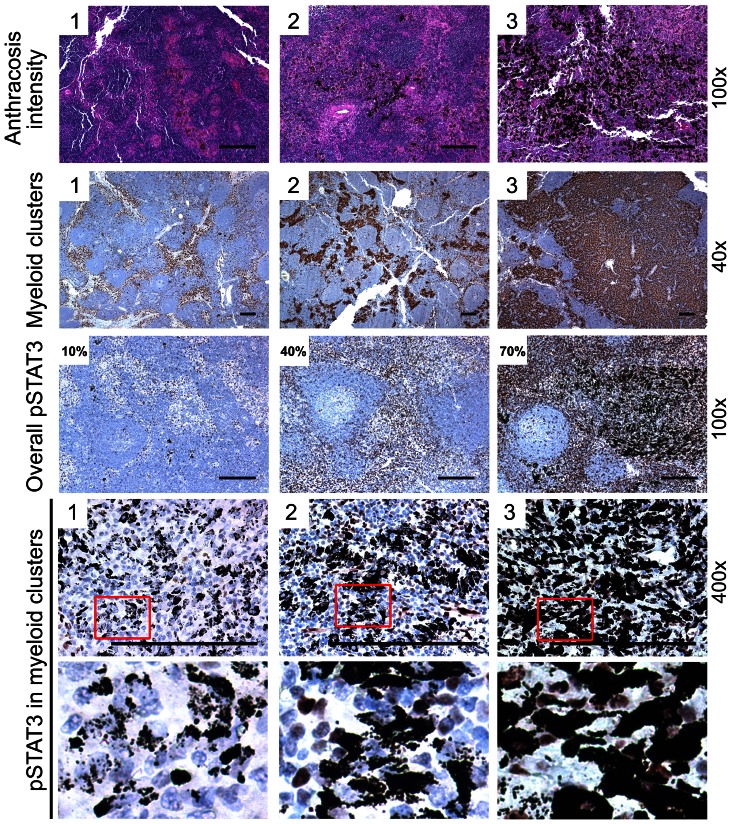
Scoring method. HE staining and IHC staining for CD68 or pSTAT3 showing representative images for the scoring of anthracosis intensity, anthracotic myeloid cluster infiltration, overall STAT3 activity and anthracotic myeloid cell STAT3 activity. The number on the upper-left corner of each image shows the score. (Scale bar, 200 µm)

Slides were evaluated under the microscope by two independent observers (W.Z. and C.Y.) without knowledge of the patients' clinical details. HE-stained sections were examined throughout under 100x magnification to score anthracosis intensity on an integer scale ranging from 1 to3 as follows: 1, small anthracotic pigment scattered in the cells and appeared cloudy; 2, carbon pigment appeared solid but scattered (not clustered); 3, clusters of more than about 50 cells with solid carbon pigment was observed. Representative images are shown in Fig. 1. For myeloid cluster infiltration evaluation, IHC slides stained for CD68 were examined under 40x magnification throughout the entire sections and scored on an integer scale from 1 to 3 as follows: 1, myeloid cluster area was smaller than 5% of the whole tissue area; 2, myeloid cluster area was between 5% and 50% of the whole LN tissue area; 3, myeloid cluster area was greater than 50% of the total LN tissue area (Fig. 1). Overall pSTAT3 level was scored as the percentage of pSTAT3^+^ cells in 10 random fields under 100x magnification (Fig. 1). STAT3 activity in myeloid cells associated with anthracosis was scored on an integer scale from 1 to 3 in 10 random fields under 200x magnification: 1, less than 5% of the anthracotic cells were pSTAT3^+^; 2, 5%–50% were pSTAT3^+^; 3, over 50% were pSTAT3^+^ (Fig. 1). In case of disagreement regarding anthracosis intensity, myeloid cluster infiltration and myeloid cell pSTAT3 level, the sections were re-evaluated by the two observers together and a final decision was made. In case of disagreement on overall pSTAT3 level, a mean value was calculated as the final score.

For prognostic evaluation, patients were dichotomized into low and high groups for each marker: Anthracosis intensity: 1 or 2 =  low, 3 =  high; myeloid cluster infiltration associated with anthracosis: 1 or 2 =  low; 3 =  high; Overall STAT3 activity: less than or equal to 50% =  low, more than 50% =  high; STAT3 activity in myeloid cells associated with anthracosis: 1 =  low, 2 or 3 =  high.

Slides stained for pan-CK using IHC were evaluated for the presence of occult metastasis under 100× and 200× magnification. Occult metastatic NSCLC cells are relatively large in size, high in nuclear-cytoplasmic ratio, and show clear and intensive ring-like cytoplasmic dark brown 3,3′-Diaminobenzidine-staining. [28]

### Cell culture

THP-1 was obtained from American Type Culture Collection and differentiated into macrophages by phorbol 12-myristate 13-acetate stimulation. Human peripheral blood mononuclear cells from healthy donors were isolated with Histopaque 1077 (Sigma). Monocytes were enriched with EsaySep CD14^+^ selection kit (StemCell Technologies) and differentiated into macrophages by culturing at presence of 10 ng/mL granulocyte-macrophage colony-stimulating factor (PeproTech) for 6 days and resting for 1 day. Macrophages were treated with nicotine (Sigma-Aldrich) with or without 1 µM AZD1480 (provided by AstraZeneca), for the indicated time.

### Western blotting

Equal amounts of cell lysate proteins prepared with RIPA buffer were subjected to sodium dodecyl sulfate polyacrylamide gel electrophoresis and immunoblotted with antibodies to pY705-STAT3 (Cell Signaling), total STAT3 (Santa Cruz) and β-actin (GE healthcare).

### Statistical analysis

Quantified positive staining areas of IHC were analyzed with Student's *t*-test. Pairwise correlations among laboratory biomarker measurement were assessed by the Spearman's rank correlation coefficient. Kaplan-Meier curves were generated to evaluate differences between survival curves and P-values based on the log-rank test were presented. Measurements of each biomarker were fit with a univariate Cox proportional hazards regression model. To investigate additional prediction power beyond clinical variables, multivariate Cox regression analysis was also carried out for model selection among stage, nodal stage, anthracosis intensity, myeloid clusters associated with anthracosis, overall pSTAT3 level, myeloid cluster pSTAT3 level and occult metastasis. An effect was considered to be statistically significant when the corresponding P-value <0.05 (*, P<0.05; **, P<0.01; ***, P<0.001). All statistical analyses were performed using the R software.

## Results

### Myeloid clusters are prominent and overall STAT3 activity is dramatically elevated in uninvolved NSCLC regional lymph nodes

**Figure 2:**
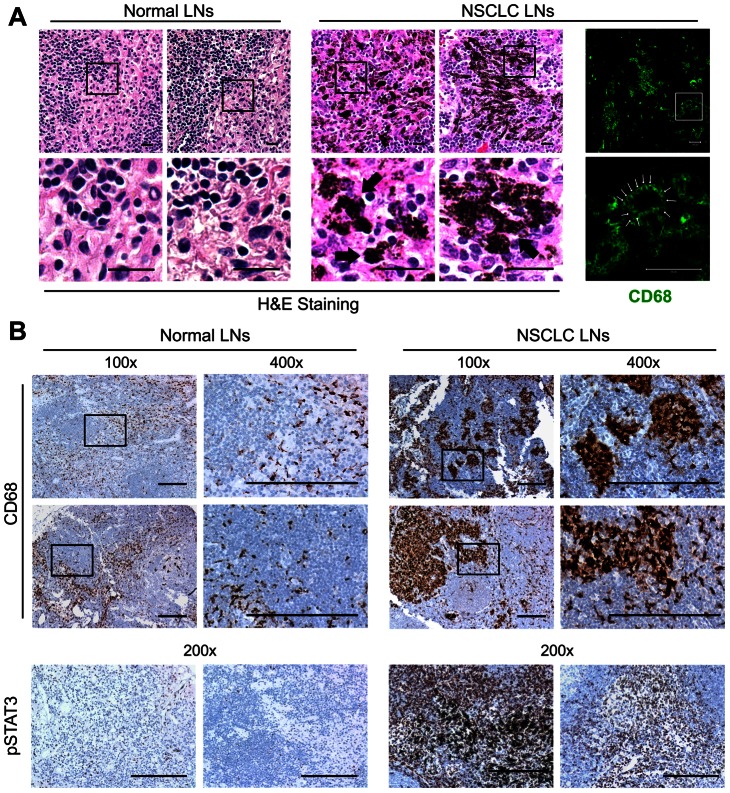
Prominent myeloid clusters associated with anthracosis and elevated STAT3 activity in NSCLC uninvolved LNs. (A) HE staining of normal LNs from individuals without cancer (n = 4; left two panels) and uninvolved regional LNs from NSCLC patients (middle two panels) showing anthracotic carbon pigment (indicated with arrows; Scale bar, 20 µm) in patient LNs. Immunofluorescent staining for CD68 (right panel) demonstrating anthracotic pigment is within CD68^+^ cells (Note the black dots indicated with small arrows in CD68^+^ cells; Scale bar, 20 µm). (B) IHC staining showing prominent CD68^+^ myeloid clusters associated with anthracosis and highly activated STAT3 in uninvolved NSCLC regional LNs (right panels) compared to normal LNs (n = 4; left panels) (Scale bar, 200 µm).

Compared with normal LNs from individuals without cancer, prominent carbon pigment deposition, i.e. anthracosis, was observed in uninvolved LNs from NSCLC patients as shown in Fig. 2A. Immunofluorescent staining demonstrated the anthracotic cells were CD68^+^ myeloid cells (Fig. 2A). Compared to uninvolved regional LNs from prostate cancer, melanoma or breast cancer patients (data not shown), the presence of myeloid cells associated with anthracosis was unique in uninvolved NSCLC regional LNs. By IHC staining, we found that the myeloid cells associated with anthracosis usually formed clusters, which was not observed in normal LNs (Fig. 2B). Elevated overall STAT3 activity in uninvolved LNs from NSCLC patients was also detected by IHC staining for pY705-STAT3 (Fig. 2B).

### Positive correlations between anthracosis intensity, myeloid cluster infiltration and pSTAT3 level in NSCLC patients with a history of smoking

**Figure 3:**
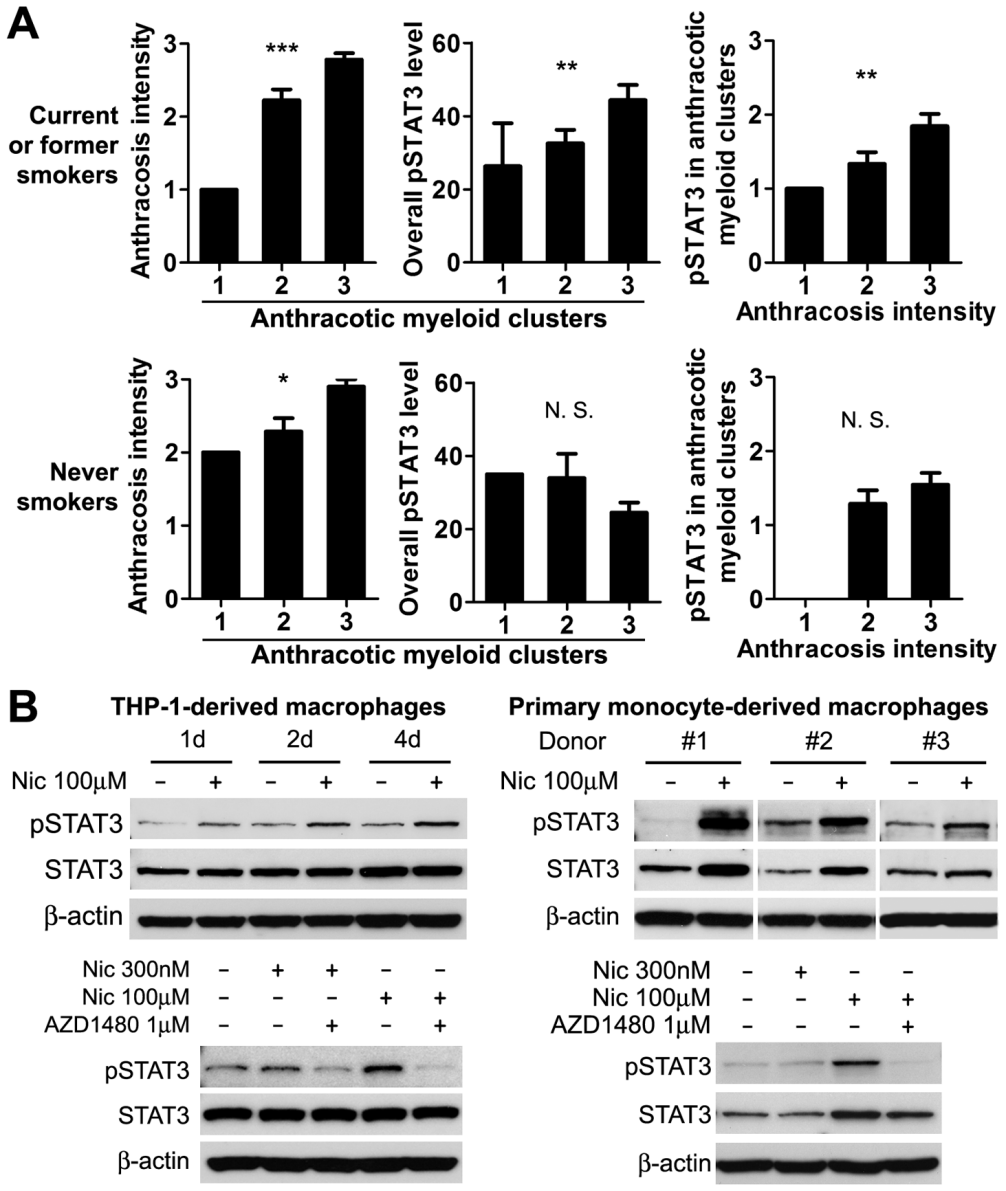
Positive correlations between myeloid clusters and STAT3 activity in patients with a smoking history. (A) In patients with a smoking history, but not in those without a history of smoking, myeloid cluster infiltration associated with anthracosis correlates with anthracosis intensity and overall STAT3 activity; anthracosis correlates with STAT3 activity in myeloid clusters associated with anthracosis. The correlation was determined with nonparametric Spearman's rank correlation test. Shown are means ± SEM, ** P<0.01, *** P<0.001. (B) Nicotine activates STAT3 in human macrophages. Western blotting showing pSTAT3 and total STAT3 expression in human THP-1-derived macrophages and human primary monocyte-derived macrophages after nicotine treatment with or without AZD1480.

In patients with a history of smoking, anthracosis intensity, myeloid clusters associated with anthracosis, overall STAT3 activity and anthracotic myeloid cell STAT3 activity were not associated with disease progression (data not shown). However, CD68^+^ myeloid cluster infiltration positively correlated with anthracosis intensity and overall pSTAT3 activity (Fig. 3A). STAT3 activation in anthracotic myeloid clusters also associated with anthracosis intensity (Fig. 3A). In contrast, in patients without a history of smoking, anthracosis was detected but correlations between STAT3 and anthracosis or myeloid clusters associated with anthracosis were not found (Fig. 3A). Thus, we focused our investigation on the role of myeloid clusters and STAT3 in the patients with a smoking history.

### Nicotine activates STAT3 in human macrophages

Tobacco smoking is one of the major causes of anthracosis within lung tissue.[4], [29] Since the correlation between anthracosis intensity and STAT3 activity was only observed in NSCLC patients with a history of smoking, we evaluated whether the addictive component of tobacco smoke, nicotine, activates STAT3 in human macrophages. As shown in Fig. 3B, STAT3 activity was elevated in both monocytic cell line THP-1-derived macrophages and primary monocyte-derived macrophages after nicotine treatment for 2 days, and the activation was completely abrogated by a Janus kinase (JAK) inhibitor, AZD1480, showing the activation is via JAK-STAT3 pathway.

### Pro-cancer and pro-metastatic phenotype of myeloid clusters

**Figure 4:**
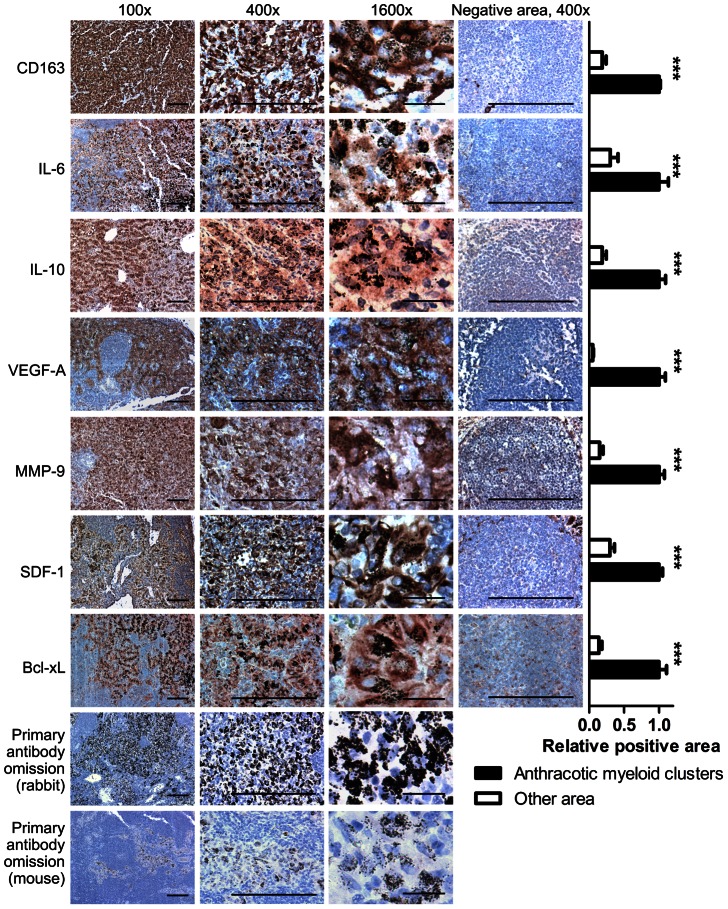
Tumor-promoting phenotype of the myeloid clusters. IHC staining demonstrating the expression of CD163, IL-6, IL-10, VEGF-A, MMP-9, SDF-1 and Bcl-xL by the myeloid cells associated with anthracosis. For each protein, representative images showing positive and negative staining areas were selected from the same slide. The quantification was performed by analyzing random images of myeloid cluster areas associated with anthracosis and other areas (10 images for each group) from 10 patients. Two-tailed Student's *t*-test was used for statistical analysis. Shown are means ± SEM, *** P<0.001. (Scale bar, 200 µm for 100× and 400×; 25 µm for 1600×)

We characterized the phenotype of CD68^+^ myeloid clusters associated with anthracosis in uninvolved regional LNs from NSCLC patients with smoking history with IHC (Fig. 4). Since anthracotic pigment stays on the tissue during immunostaining, it serves as a cytoplasmic marker for the myeloid cells associated with anthracosis. The expression of CD33 confirmed these cells were from myeloid linage (data not shown). To investigate whether the myeloid clusters were M2-polarized, we performed CD163 staining and the clusters showed strong staining. Investigation of myeloid cluster phenotype showed expression of IL-6 and IL-10, which are both immunosuppressive factors. The expression of VEGF-A and MMP-9 indicated a role in angiogenesis and tissue-remodeling. VEGF-A is also known to inhibit dendritic cell maturation.[30] Activation of STAT3 up-regulates Bcl-xL expression in myeloid cells,[24] and myeloid clusters showed positive staining for Bcl-xL. SDF-1/CXCL12 has a role in recruiting NSCLC via the SDF-1/CXCL12-CXC chemokine receptor type 4 axis,[31], [32] and IHC staining showed that SDF-1 was highly expressed by myeloid clusters. All these molecules are STAT3 downstream regulated genes.[7] In addition, IL-6, IL-10 and VEGF-A are STAT3 activators.[7] Statistical analysis showed a significant difference in the expression of these molecules between myeloid clusters associated with anthracosis and other areas within the LN.

### Myeloid clusters correlate and colocalize with LN occult metastasis

**Figure 5:**
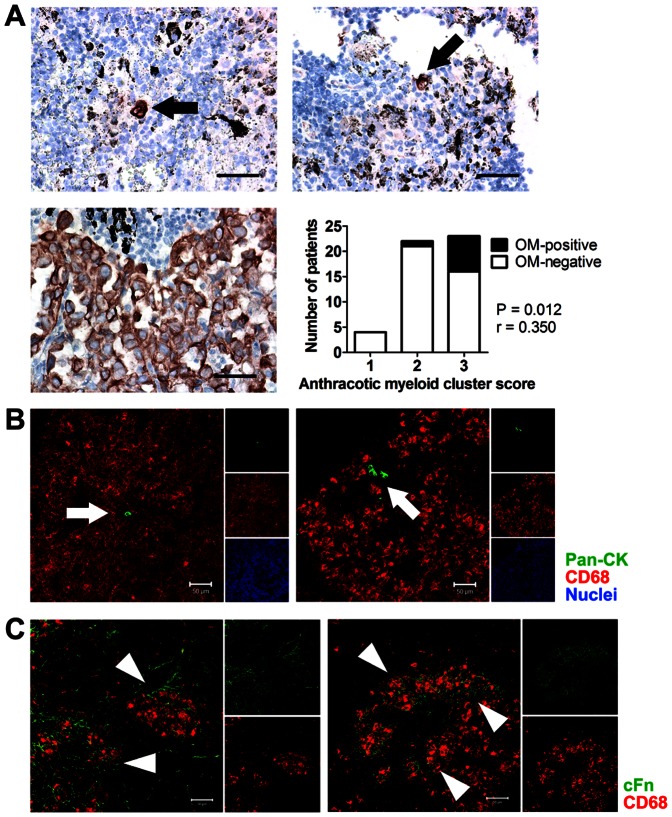
Myeloid clusters in uninvolved LNs colocalize with occult metastasis and cFn. (A) IHC staining showing occult metastasis in “uninvolved” LNs (top two panels) and metastatic tumor cells in an involved LN as the positive control (lower left panel) (Scale bar, 50 µm). The bar graph shows the correlation between myeloid cluster infiltration associated with anthracosis and the presence of occult metastasis (OM). The P value was determined by nonparametric Spearman's rank correlation test. (B) Confocal images showing the colocalization of pan-CK^+^ NSCLC cells (arrows) and CD68^+^ myeloid clusters associated with anthracosis. (Scale bar, 50 µm) (C) Confocal images showing the colocalization of myeloid clusters associated with anthracosis (arrows) and cFn. (Scale bar, 50 µm)

The presence of occult LN metastasis has been linked to poor prognosis in a large cohort of patients with resectable NSCLC.[28] By evaluating pan-CK, we detected occult metastatic tumor cells in the LNs of 8 patients out of the 49 patients with a smoking history (Fig. 5A). Interestingly, the majority of patients positive for occult LN metastasis (7 out of 8) showed increased myeloid clusters associated with anthracosis in the uninvolved LNs (Fig. 5A). The presence of occult LN metastasis was positively correlated with myeloid clusters associated with anthracosis (P = 0.012, r = 0.350) and anthracosis intensity (P = 0.032, r = 0.310). In addition, the occult metastatic tumor cells were usually detected within or very close to clusters of myeloid cells associated with anthracosis (6 out of 8; Fig. 5A(a–b)). Double staining for pan-CK and CD68 confirmed the colocalization (Fig. 5B).

It has been shown that cFn expression in pre-metastatic tissue is elevated and it plays an important role in the formation of pre-metastatic myeloid clusters.[16] In addition, STAT3 regulates the expression of cFn in pre-metastatic myeloid cells.[24] LN sections stained for cFn and CD68 showed that cFn is colocalized with small clusters of myeloid cells associated with anthracosis (Fig. 5C), indicating a possible role of cFn in the recruitment of myeloid cells and cluster formation in NSCLC regional uninvolved LNs.

### Myeloid clusters and overall pSTAT3 level predict the prognosis of NSCLC patients with a history of smoking

**Figure 6:**
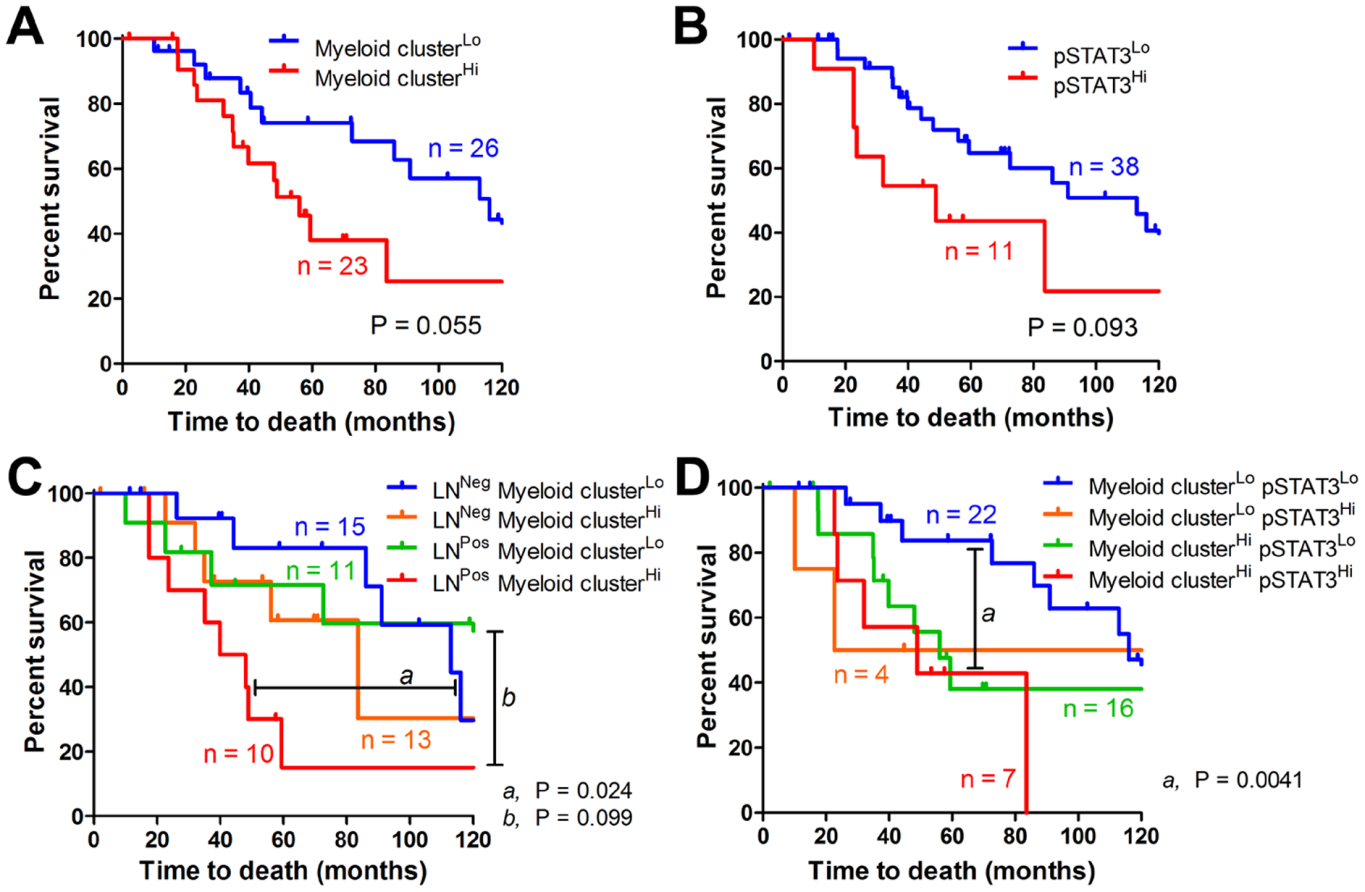
Myeloid clusters and pSTAT3 predicts survival in NSCLC patients with a smoking history. Kaplan-Meier curves showing the survival of patients grouped by: (A) myeloid cluster infiltration associated with anthracosis, (B) overall pSTAT3 level, (C) the combination of nodal stage and myeloid clusters associated with anthracosis, (D) the combination of myeloid clusters associated with anthracosis and overall pSTAT3 level (n = 49).

We assessed the prognostic value of anthracosis intensity, CD68^+^ myeloid cluster infiltration associated with anthracosis and pSTAT3 level. In a univariate analysis, anthracotic myeloid cluster score showed a trend toward significance in the prediction of overall survival (P = 0.061, Hazard ratio (HR) = 2.22, 95% CI [0.96, 5.13]). A Kaplan-Meier survival estimate with Log-rank test also demonstrated a near-significant difference between myeloid cluster associated with anthracosis-high and -low group (P = 0.055; Fig. 6A). Both median survival and 5-year survival are improved in the low myeloid cluster group (median survival: 116.1 months, 95% CI [86.0, NA] vs. 56.1 months, 95% CI [35.1 - NA]; 5-year survival: 78.5%, 95% CI [63.3%, 97.3%] vs. 38.0%, 95% CI [20.8%, 69.3%]). Moreover, overall pSTAT3 showed a trend toward significance in the prediction of overall survival (P = 0.093; Fig. 6B).

In a multivariate Cox regression analysis for model selection,, nodal stage and anthracotic myeloid cluster score were left in the final model (nodal stage: P = 0.033, HR = 1.75, 95% CI [1.05, 2.94]; anthracotic myeloid cluster score: P = 0.049, HR = 2.34, 95% CI [1.00, 5.43]), showing that myeloid clusters associated with anthracosis in regional uninvolved LNs provide additional prediction power to patient overall survival beyond conventional clinicopathological variables. Therefore, we combined nodal stage and myeloid clusters associated with anthracosis to improve the overall survival prediction. Despite a limited sample size, patients with negative LNs and low myeloid clusters demonstrated significantly improved survival compared to those with positive LNs and high myeloid clusters (P = 0.024; median survival: 113.0 months vs. 44.0 months; 5-year survival: 83.1% vs. 15.0%; Fig. 6C). The combination of anthracotic myeloid cluster score and overall pSTAT3 level also defined two populations of patients with significantly different prognosis (P = 0.0041; Fig. 6D). These data suggest that myeloid clusters associated with anthracosis in uninvolved LNs are independent predictors of poor prognosis in smoking NSCLC patients.

## Discussion

Development of metastatic disease following surgical resection of NSCLC requires multifaceted interactions facilitated by tumor and stromal cells within the microenvironment at the metastatic site, including immune cells. Infiltration of CD68^+^ myeloid cell clusters associated with anthracosis was prominent in benign LN from NSCLC patients and activation of STAT3 was elevated, consistent with earlier reports in multiple malignancies.[14], [24] Pre-metastatic niche formation has been attributed to a variety of mediators including VEGF, MMP-9, STAT3 and SDF-1/CXCL12.[16], [20], [21], [24] Phenotypic delineation of the myeloid clusters seen in uninvolved LN showed expression of IL-6, IL-10 and VEGF-A as inhibitors of antitumor immunity, VEGF-A and MMP-9 as pro-angiogenic factors and SDF-1 as a chemoattractant to circulating NSCLC cells. The abundant myeloid clusters associated with anthracosis in uninvolved LNs may contribute to the immunosuppressive environment and play a pro-cancer and pro-metastatic role. Expression of anti-apoptotic gene Bcl-xL is also consistent with a previous report on pre-metastatic myeloid clusters.[24]

In the tumor microenvironment, STAT3 mediates multidirectional crosstalk between tumor-associated myeloid cells and other stromal cells, including endothelial cells. [10] Thus, in addition to the effect of tumor-secreted factors and smoking, the elevation of STAT3 activity in pre-metastatic LNs may also contribute to tumor metastasis and progression through the infiltration of myeloid cells. Recent investigations demonstrate that inhibition of STAT3, which is constitutively activated in pre-metastatic niches, can decrease myeloid cell infiltration in future metastatic sites.[24], [33] STAT3 also impairs antitumor immunity to facilitate carcinogen-induced lung tumor formation.[34] Our data shows that nicotine was able to activate STAT3 in human macrophages with subsequent abrogation by JAK/STAT inhibition, suggesting that formation of pre-metastatic sites and metastases in patients with smoking-related NSCLC may be sensitive to STAT3 blockade.

Detection of occult metastases in patients with resected NSCLC provides additional prognostic criteria, which may be used in determining adjuvant therapy.[28] When we evaluated occult metastasis in benign LNs, most were found in those with high levels of myeloid clusters associated with anthracosis and the occult metastatic tumor cells colocalized within these clusters. Recruitment of these myeloid clusters and subsequent occult metastasis may be due to cFn, which was also present in uninvolved LN at the site of the clusters.

Because myeloid cell STAT3 activity not only impacts immune regulation in the context of tumor,[14] but also plays an important role in pre-metastatic tissue,[24] the formation of myeloid clusters and the activation of STAT3 may affect patient prognosis. In patients without lymph node involvement, those with low levels of myeloid clusters associated with anthracosis showed improved survival. The combination of anthracitic myeloid cluster score and pSTAT3 level further defined prognosis beyond tumor and LN stage in patients with resectable NSCLC.

Although our findings are promising, the current study has several limitations. First, the conclusions are limited by the small number of patients that form our cohort. Future studies with larger patient numbers will be performed to validate the conclusions. Second, our previous publication showed that STAT3 activation in myeloid clusters is crucial for their colonization at pre-metastatic sites to facilitate metastasis in mouse models. [24] Likewise, our current study indicates that myeloid cluster infiltration in uninvolved LNs of NSCLC patients correlates with occult metastasis and prognosis. However, it is important to note that the correlation does not equal causation. Myeloid cluster accumulation in NSCLC pre-metastatic LNs may occur as a secondary event during metastatic progression. Thus, direct evidence would be necessary to prove that CD68^+^ myeloid clusters are responsible for priming NSCLC nodal metastasis.

Despite the limitations, our study provides evidence that STAT3 and myeloid infiltration associate with early metastatic disease and may be important in smoking-induced lung cancers. The correlation of myeloid clusters associated with anthracosis and pSTAT3 with patient survival offers additional information when considering future therapy. Inhibition of pathways which stimulate pre-metastatic niche formation may prove more effective than standard chemotherapy. Investigation in larger cohorts for validation and prospective trials will further define the role of myeloid clusters and STAT3 in early stage NSCLC. Furthermore, examination of fresh tissue and isolation of viable myeloid clusters will provide insights into the cellular mechanisms involved.
